# Poisoned Wounds from the Bite of the Snake and Tarantula, and the Sting of the Scorpion and Centipede

**Published:** 1872-10

**Authors:** James S. Bailey

**Affiliations:** Albany, N. Y.


### Poisoned Wounds from the bite of the Snake and the Tarantu'a and the Sting of the Scorpion and Centipede.

By James S. Bailey, M. D., Albany, N. Y.

While perusing "Emergencies, and How to Treat Them," by Dr. Howe, my attention was directed to chapter VI., which treats upon poisoned wounds. After discussing dissection wounds, and hydrophobia, he refers to snake and tarantula bites, also to the sting of the scorpion and centipede.

Physicians commencing the practice of medicine eagerly adopt remedies recommended by authors: therefore the necessity of correct teaching, or much harm may result. From his remarks it is to be supposed that his personal experience is these "Emergencies" has been limited, and that he has gathered his ideas from books.

There is great difference between theory and practice in the treatment of bites of venomous serpents and insects, and few emergencies require more prompt and active treatment. By the timely administration of efficient remedies the mortality can be much reduced, and if energetic treatment is not pursued the life of the victim may be sacrificed.

While living in the extreme South, I frequently had occasion to treat accidents of this kind, and did not deem it prudent to experiment, but relied upon remedies known to be efficacious.

My chief reliance was upon suction and alkaline dressings; soda or ammonia applied externally, with large draughts of spirituous liquors, whiskey or brandy, internally; or full and repeated doses of olive oil taken into the stomach. These remedies were found to be certain and speedy antidotes. I have never known a person to die from a snake-bite who could be gotten under the influence o liquor, and the success of the internal use of olive oil is equally efficacious.

A domestic remedy was frequently applied before a physician could be summoned; which was to kill a chicken, open it through the back, and apply it warm with its entrails over the wound. Its efficacy was evident by the parts of the fowl coming in contact with the wound in a few minutes turning green by absorbing the poison.

When a fang of the serpent penetrates a blood-vessel and the virus is absorbed directly into the circulation, remedies avail but little, and the person usually lives but a short time; but if such is not the case, the timely administration of appropriate remedies affords relief.

Swine are not impressed by snake-bites, because of the deposit of adipose next the skin, which neutralizes the poison, and besides it is not abundantly supplied with blood-vessels. With lean hogs it is very different. I have frequently noticed a lean hog to avoid a poisonous reptile, while one in good flesh would attack eagerly and devour without fear. The instinct of this brute is such, which seemingly is endowed with but limited reasoning power.

The scorpion sting is never dangerous, though exceedingly painful, even more so than the sting of a bee, but the pain does not last so long. Treatment is unnecessary, though alkalies applied directly relieve.

The tarantula bite is very dangerous and painful. The treatment suggested for snake-bite is appropriate, if resorted to at once. I have never known a fatal case, though extensive sloughing has sometimes taken place.

Dr. Howe remarks: "The stories of its ravages are however not founded upon facts." Could the Dr. have seen some of my cases he would have thought differently.

He again remarks: "Centipedes are less dangerous than either of the preceding varieties."

This is not in accordance with my experience; the mentioning of a case will illustrate.

A gentleman in high social position, while engaged in superintending his garden, drew of his coat and hung it upon a shrub which by its weight bent it to the ground; a centipede crawled up one sleeve; upon putting on his coat he felt a creeping sensation upon his arm: he squeezed the part and attempted to brush it down, when each poisoned claw made its impress upon the arm, and in consequence he was soon in great agony.

The ordinary domestic remedies were immediately applied before a physician arrived, but in spite of treatment a high grade of inflammation was kindled; suppuration and sloughing took place, destroying the surrounding tissues, which left the arteries pulsating plainly visble and well nigh destroyed the man's life. There eventually was much loss of substance of the affected arm. In the course of three months he was measurably restored to health.

Poultry greedily devour the smaller specimens of the tarantula with impunity, but the virous from its sting is a deadly poison to the human subject.

Fortunately, wounds from the tarantula and centipede seldom occur, but snake-bites are more common where poisonous reptiles abound.

Dr. Tanner remarks, in his "Practice of Medicine," that dengue fever is an accompaniment of scarlet fever. We that have treated dengue fever laugh at this statement, and the trusting to bilious antidotes in the emergencies of snake-bites seems equally absurd to physicians having much experience in such cases.—*Medical Record.*

